# Evaluation of Polyvinyl Alcohol/Cobalt Substituted Hydroxyapatite Nanocomposite as a Potential Wound Dressing for Diabetic Foot Ulcers

**DOI:** 10.3390/ijms21228831

**Published:** 2020-11-22

**Authors:** Wei-Chun Lin, Cheng-Ming Tang

**Affiliations:** 1 School of Dental Technology, College of Oral Medicine, Taipei Medical University, Taipei 110, Taiwan; tukust94114wenny@gmail.com or; 2 Graduate Institute of Oral Sciences, Chung Shan Medical University, Taichung City 40201, Taiwan; 3 Department of Dentistry, Chung Shan Medical University Hospital, Taichung City 40201, Taiwan

**Keywords:** polyvinyl alcohol, cobalt-substituted hydroxyapatite, diabetic foot ulcers, hydrogels, antibacterial ability

## Abstract

Diabetic foot ulcers (DFUs) caused by diabetes are prone to serious and persistent infections. If not treated properly, it will cause tissue necrosis or septicemia due to peripheral blood vessel embolism. Therefore, it is an urgent challenge to accelerate wound healing and reduce the risk of bacterial infection in patients. In clinical practice, DFUs mostly use hydrogel dressing to cover the surface of the affected area as an auxiliary treatment. Polyvinyl alcohol (PVA) is a hydrophilic hydrogel polymer widely used in dressings, drug delivery, and medical applications. However, due to its weak bioactivity and antibacterial ability, leads to limited application. Filler adding is a useful way to enhance the biocompatibility of PVA. In our study, cobalt-substituted hydroxyapatite (CoHA) powder was prepared by the electrochemically-deposited method. PVA and PVA-CoHA nanocomposite were prepared by the solvent casting method. The bioactivity of the PVA and composite was evaluated by immersed in simulated body fluid for 7 days. In addition, L929 cells and *E. coli* were used to evaluate the cytotoxicity and antibacterial tests of PVA and PVA-CoHA nanocomposite. The results show that the addition of CoHA increases the mechanical properties and biological activity of PVA. Biocompatibility evaluation showed no significant cytotoxicity of PVA-CoHA composite. In addition, a small amount of cobalt ion was released to the culture medium from the nanocomposite in the cell culture period and enhanced cell growth. The addition of CoHA also confirmed that it could inhibit the growth of *E. coli*. PVA-CoHA composite may have potential applications in diabetic trauma healing and wound dressing.

## 1. Introduction

Diabetes is an endocrine disease caused by abnormal sugar and fat metabolism. It often causes other complications such as vascular disease, nephropathy, and diabetic foot ulcers (DFUs). Among them, DFUs are caused by poor long-term blood glucose control, which causes lower limb peripheral arterial disease and foot deformation [1]. When ulcers occur without proper treatment, it will cause tissue necrosis or amputation due to peripheral blood vessel embolism. In addition, bacteria can also spread through the blood circulation to cause septicemia and threaten the patient’s life. Therefore, it is an urgent challenge to accelerate the speed of wound healing and reduce the risk of bacterial infection in patients. In clinical practice, dressing is often used to cover the surface of the affected area as one of the treatment methods for DFUs. The ideal wound dressing needs to have the following conditions: (1) can provide gas and liquid exchange to keep the wound properly moist [2,3]. (2) It has favorable biocompatibility and does not cause any immune response. (3) Protects wounds to reduce infection and (4) the dressing is easily removed and does not cause secondary trauma [4,5]. Based on the above conditions, hydrogel-like materials are widely used in wound dressing.

Polyvinyl alcohol (PVA) has excellent film forming properties and outstanding chemical stability, widely used in industry applications such as food packaging [6]. In addition, PVA has non-toxic and low protein adsorption properties, resulting in low cell adhesion compared to other hydrogel-like materials [7]. Therefore, it is developed in artificial cartilage substitutes, corneal implants [8], and drug delivery [9]. However, PVA is relatively limited in terms of its bioactivity compared to other polymers. On the other hand, nano-scale particles widely added into polymer to enhance its mechanical properties and thermal properties is a common modification procedure [10], while the effect of nano-scale particle additives is much more remarkable than that observed in conventional composites because of the high surface to volume ratio of the nano-scale particles. For example, the mechanical and thermal properties of PVA were outstanding, improved upon the addition of a small amount (<5%) of bamboo charcoal nanoparticles (107.4 ± 9.8 nm) [11].

Furthermore, hydroxyapatite (HA) is the main component of human bones and the calcium atom in the apatite inner structure can be substituted by divalent metal cations [12,13,14], altering the original properties of HA such as the lattice, crystallinity, and grain shape [15]. In addition, cobalt ion is a hypoxia-mimicking agent, which can activate the hypoxia inducible factor-1 (HIF-1α) in bone marrow stromal stem cells and subsequently activate HIF-1α target genes including erythropoietin and vascular endothelial growth factor (VEGF) [16,17,18,19]. This indicates that cobalt ions have the property of inducing angiogenesis. For problem wounds caused by diabetes, in addition to reducing wound infections, the supply of nutrients will also affect wound repair. Therefore, cobalt ions can be used to promote angiogenesis to help transport nutrients to the wound to help repair. Previous literature shows that synthesized cobalt-substituted hydroxyapatite (CoHA) (Co^2+^ content 12 wt%) can enhance osteogenesis in osteoporosis-induced alveolar defects after 24 weeks [20]. Our previous studies have confirmed that the release of cobalt ions through CoHA can promote bone cell growth, reduce free radicals, and enhance antibacterial effects [21,22]. This shows that CoHA is a potential choice for wound dressing additives.

According to the above, this study involves the preparation of PVA/CoHA composites membranes. The main objectives are to analyze the effect of CoHA on the properties of PVA and on improving the biocompatibility, anti-bacterial ability, and anti-inflammatory response of the membrane. We hypothesized that the addition of CoHA is significantly effective for the bioactivity and anti-inflammatory properties of the membranes. Finally, we assess whether the composite membrane could be used for the application of diabetic traumas.

## 2. Results and Discussion

### 2.1. Characterization of CoHA

**Figure 1:**
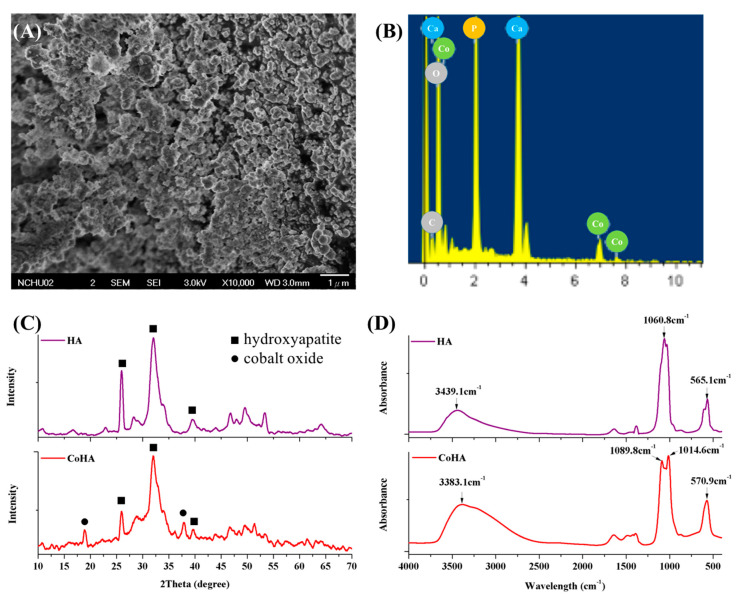
(**A**) Field emission scanning electron microscopy (FESEM) image of cobalt-substituted hydroxyapatite (CoHA) powder, (**B**) surface element analysis of CoHA by energy dispersive X-ray spectroscopy (EDS), (**C**) X-Ray diffraction (XRD) patterns of hydroxyapatite (HA) and CoHA, (**D**) IR spectra of HA and CoHA by FTIR.

The surface morphology of CoHA shows a granular mixed structure (Figure 1A). It is evident that most of the nanoparticles are agglomerated. The surface elemental composition of CoHA was analyzed using energy dispersive X-ray spectroscopy (EDS) and cobalt elements were found (Figure 1B). The crystal structure of the powder was analyzed by XRD, diffraction peaks of HA crystals were observed at 26.01° (002), 32.04° (211), 39.68° (310), 46.81° (222), and 49.74° (123) and diffraction peak of Co_3_O_4_ was observed at 18.8° (111), which confirmed the presence of the CoHA structure (Figure 1C). The chemical compositions of HA and CoHA were obtained by FTIR. The absorption peaks at wavenumbers of 570.9 cm^−1^ represent ${\mathrm{PO}}_{4}^{3-}$. The peak at 3383 cm^−1^, representing OH^−^ is observed in HA and CoHA, among which CoHA showed the largest peak shift (Figure 1D). This could be caused by the addition of CoCl_2_, which was combined with OH^−^ to observe the peak shift [21].

### 2.2. Characterization of PVA Nanocomposite

**Figure 2:**
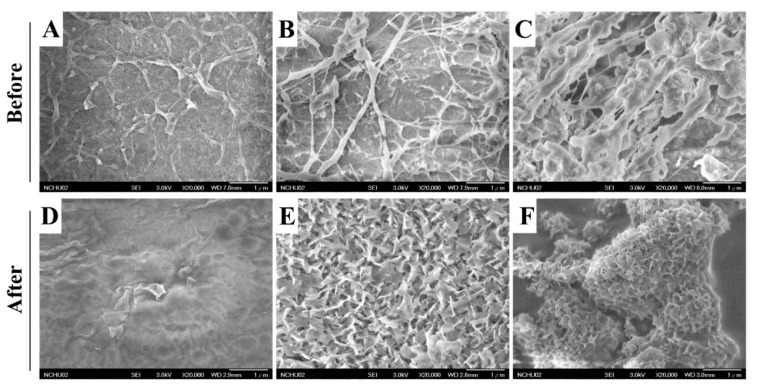
Surface morphology of (**A**,**D**) PVA, (**B**,**E**) PVA-HA nanocomposites, and (**C**,**F**) PVA-CoHA nanocomposites before and after immersion in SBF for 7 days.

Dried PVA and nanocomposite membranes had a smooth surface (data not shown). When the membranes were continuously immersed in distilled water at 37 °C for 24 h, the overall structure retains its integrity. However, swollen membranes were dehydrated and an interconnected porous structure on the surface was observed by FESEM (Figure 2A–C).

**Figure 3:**
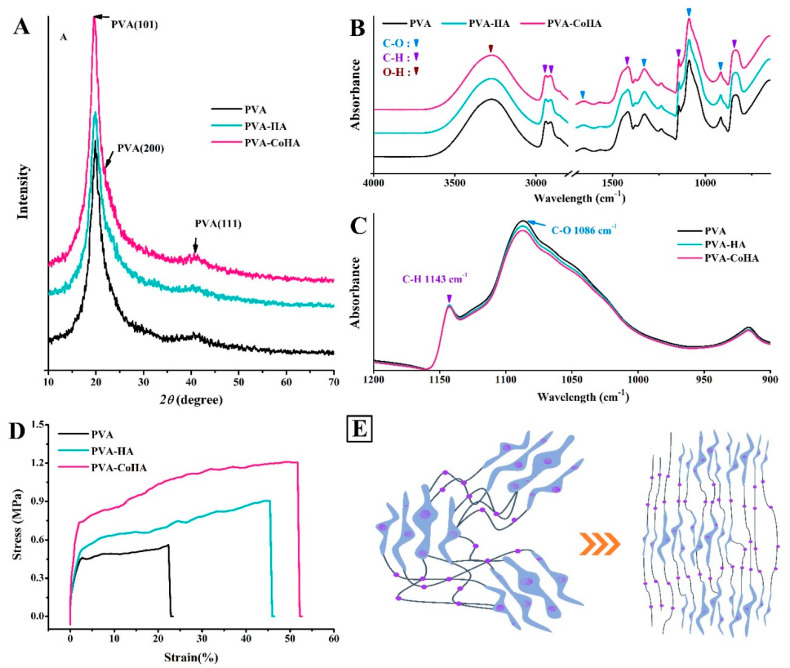
(**A**) XRD patterns, (**B**,**C**) ATR-FTIR spectra, and (**D**) stress–strain curve of PVA and PVA-CoHA nanocomposites. (**E**) Structure model for extended PVA-CoHA.

The crystal structure is one of the major factors that affect the mechanical properties of a material. The XRD patterns of the PVA and PVA/CoHA nanocomposites are presented in Figure 3A. The XRD pattern of the pure PVA membranes revealed strong crystalline reflections at around 2θ = 19.88° and a shoulder at 22.74°. The two peaks are characteristic of PVA, representing reflections from (101) and (200) from a monoclinic unit cell [23]. In the XRD profile of PVA/Co-HA nanocomposite membranes, no peak from Co-HA was observed in the XRD curves of the nanocomposites [11,24].

ATR-FTIR analysis was conducted on the PVA and PVA nanocomposite membranes (Figure 3B,C). PVA absorption peaks appeared at 3255 cm^–1^ (stretching of OH), 2941, 2906 cm^–1^ (symmetric stretching of CH_2_), 1569 cm^–1^ (O–H and C–H bending), 1416 cm^–1^ (bending of OH and wagging of CH_2_), 1658 and 1330 cm^–1^ (C = O), 1142 cm^–1^ (CH wagging), 1086 cm^–1^ (stretching of CO and bending of OH from amorphous sequence of PVA), 920 cm^–1^ (CO symmetric stretching), and 837 cm^–1^ (CH_2_ rocking) [11,24,25,26,27,28]. The small bands found at 854 cm^–1^ were generated by the stretching vibrations of C–C bonds [23]. The crystallinity of PVA was obtained from the peak at 1143 cm^–1^ in the ATR-FTIR spectrum. The peak symmetric stretching mode of the C-C or C-O stretch of the chain is related to the intramolecular hydrogen bonding between two adjacent OH groups on the same side of the carbon plane [29]. The peak at 1143 cm^−1^ indicates that C-O is stretched from the crystalline sequence of PVA, while the peak at 1086 cm^−1^ indicates C-O stretching from the amorphous sequence of PVA [30]. Therefore, the ratio of the two peaks reflects the crystallinity of PVA. The position strengths through 1143 cm^–1^/1086 cm^–1^ is calculated as surface crystallinity of PVA (%) = [Absorbance at 1143 cm^−1^/(Absorbance at 1143 cm^−1^ + Absorbance at 1086 cm^−1^)] × 100%. The results show that the addition of pure HA and CoHA slightly increases the crystallinity on the surface of the membranes (Table 1), but there is no significant difference.

Tensile testing showed that the addition of CoHA significantly increased the tensile strength and ductility of PVA (Figure 3D). This result is similar to the literature, adding a small number of nanoparticles can improve the mechanical properties of the polymer [11]. In addition, the OH group on the molecular chain of CoHA and PVA generates a hydrogen bond to restrict the movement of the molecular chain which is called the crystalline region. However, the unaffected molecular chains can move freely and are called amorphous regions. As shown in Figure 3E, the crystalline region acts to enhance mechanical strength, while the non-crystalline region imparts ductility to the film.

### 2.3. Thermal Properties

**Figure 4:**
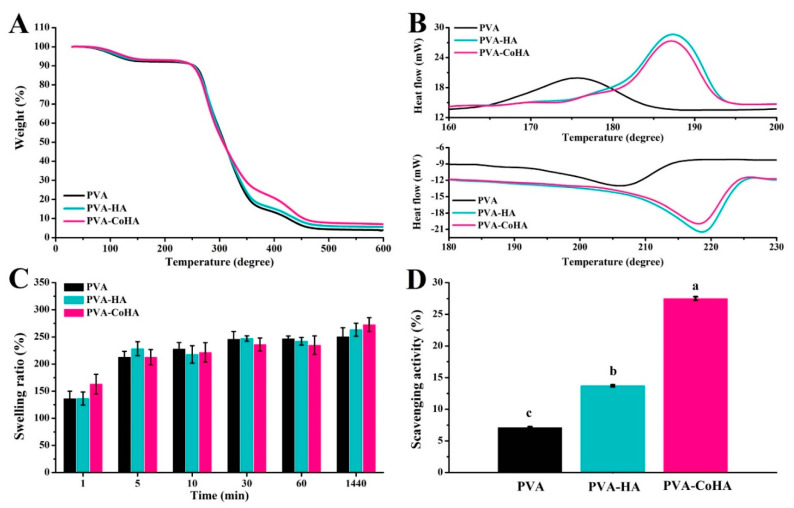
(**A**) TGA patterns, (**B**) DSC patterns, (**C**) swelling behaviors curve, and (**D**) free radical scavenging ability of PVA and PVA-CoHA nanocomposites. Means with different letters (a–c) were significantly different (*p* < 0.05, mean ± SD, *n* = 4).

Thermal properties of the PVA and nanocomposites were assessed using thermo-gravimetric analyzer (TGA) and differential scanning calorimetry (DSC) data and are summarized in Table 2. Treating PVA at 100 °C completely removed any moisture present, [24] and heating it at 247–362 °C caused rapid decomposition (Figure 4A,B). The composite film changes at 380 °C. The addition of CoHA was found to slow down the weight loss of PVA. From the SEM (Figure 2A–C) speculation, this phenomenon can be explained by the fact that PVA uniformly coats CoHA particles and makes the structure denser. It also explains that PVA-CoHA has a higher crystallinity of PVA internally. PVA-CoHA heated to 400 °C led to 20.75% residual material, significantly higher than the case of pure PVA (13.32% residual, Table 2). The ash content of PVA-CoHA at the temperature end point (600 °C) is still higher than that of pure PVA (3.08% at 400 °C), denoting that CoHA content also increases the thermal properties of PVA. The results of *X*c are also listed in Table 3. DSC results show that the addition of CoHA can significantly increase the crystallization of PVA, which is consistent with the XRD results where the typical PVA peak (101) in PVA-CoHA is the highest.

### 2.4. Swelling Behavior

Swelling property of wound dressing will affect drug release properties and interactions with tissue fluid on wound position. Therefore, swelling behavior is one of the important indicators for evaluating dressings. PVA and PVA-CoHA membranes were immersed in PBS as a model for human contact (pH = 7.42) at 37 °C, and the swelling behavior was calculated at different immersion times (Figure 4C). The results showed that PVA and PVA-CoHA nanocomposites membranes have rapid and good swelling properties (Figure 4C), where soaking in PBS for one minute leads to 150% expansion. After 24 h soaking in PBS, the membrane reached 260% expansion and an equilibrium state, and statistical analysis indicated no significant differences among the tested sample groups. Therefore, the addition of CoHA does not affect the swelling behavior of PVA and can ensure that the membrane maintains a good water absorption effect.

### 2.5. Free Radical Scavenging Ability

Anti-inflammatory effects of wound dressings have attracted more attention. Biological defense and the differentiation process will produce free radicals. However, excessive free radicals may cause excessive oxidation. Antioxidants can capture excessive harmful free radicals to maintain the normal growth of organisms. Therefore, they play an important role in this balance. Figure 4D shows the free radical scavenging ability of the PVA-Co-HA composite membrane. The capture capacity of PVA-CoHA was significantly higher than that of pure PVA or PVA-HA (*p* < 0.05). It is mainly the exposed CoHA on the surface that allows PVA-CoHA to combine with more free radicals. Therefore, it indirectly indicates that PVA-CoHA can reduce the anti-inflammatory effect of cells and reduce the inflammatory response of wounds.

### 2.6. Hydrophilicity Test

**Figure 5:**
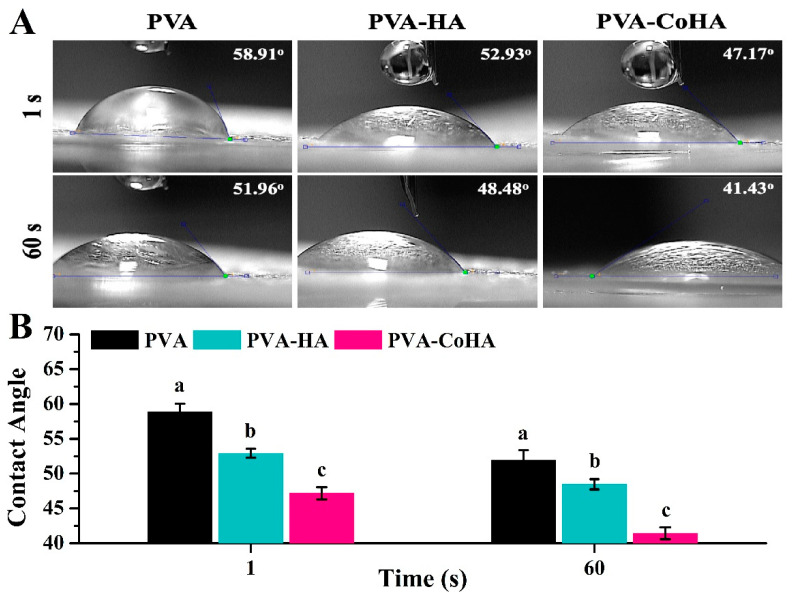
The surface wettability of PVA and PVA nanocomposites. (**A**) Image of surface contact angle. (**B**) Quantitative analysis of the contact angle was performed. Means with different letters (a–c) were significantly different (*p* < 0.05, mean ± SD, *n* = 5).

The hydrophilicity and hydrophobicity of the material surface is one of the important indicators of biomedical materials [31]. Use contact angle measurement to evaluate the hydrophilicity and hydrophobicity of the material surface to observe the effect of the material surface on cell adhesion. It is reported in the literature that increasing the hydrophilicity of the material can improve its biological activity and contribute to cell adhesion [32]. In order to evaluate the surface contact angle of the PVA membranes after contact with the skin. In this study, PBS was used as the test solution. The results show that both PVA and PVA-CoHA nanocomposites membranes are hydrophilic materials (Figure 5). In addition, the time that the solution stays on the surface is negatively correlated with the contact angle. This is similar to the result of swelling. The PVA membranes start to absorb the liquid on the surface after 1 min and reduced the contact angle. However, the surface of PVA-HA and PVA-CoHA is relatively hydrophilic due to the combination of the structural solution with OH on the surface and water molecules. Especially the SEM image can find that the exposure of CoHA in PVA-CoHA is more obvious, which leads to a significantly smaller contact angle than other groups (*p* < 0.05). The above addition of HA and CoHA can effectively improve the hydrophilicity of the PVA surface.

### 2.7. Bioactivity

Many studies use surface mineralization results to evaluate the bioactivity of materials. Surface mineralization results are given in Figure 2D–F for PVA and PVA-CoHA nanocomposites membranes placed in 37 °C SBF solution for 7 days. The pure PVA membrane surface did not produce HA at any exposure time, while PVA-HA and PVA-CoHA produced HA, especially after 7 days of SBF exposure.

**Figure 6:**
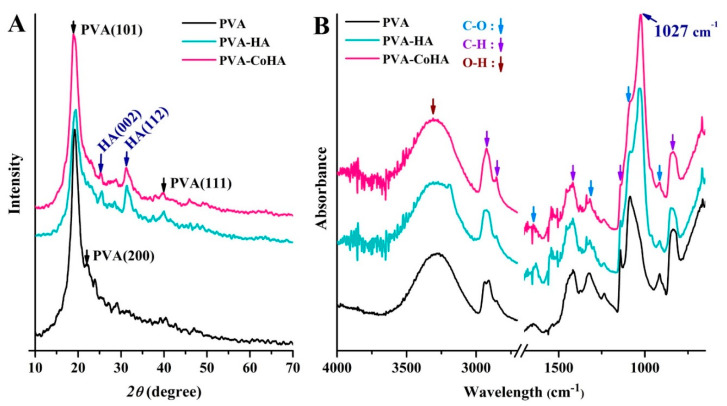
(**A**) XRD patterns and (**B**) ATR-FTIR spectra of PVA and PVA-CoHA nanocomposites after immersion in SBF for 7 days.

Based on EDS data, the Ca/*p* ratios of different sample types were 1.29 (PVA-HA) and 1.39 (PVA-CoHA). The apatite crystal faces also differ between samples, where the PVA-HA crystalline morphology is a sheet of flat HA. PVA-CoHA feature lamellae divided into regions rather than a flat surface, which may be due to the impact of cobalt ion remineralization during crystallization. Notably, there were cobalt ions before immersion in the CoHA samples, and after 7 days of SBF immersion these ions disappeared due to apatite deposition (Table 3). This indicates that the membrane surface undergoes new HA deposition, covering the cobalt ions. XRD data show that the diffraction peaks of pure PVA do not produce new peaks after SBF soaking, indicating that the membrane does not undergo mineralization (Figure 6A). HA and CoHA were added to membranes, creating a new diffraction peak at 26° and 32°. The diffraction peaks for HA are (002) and (112), respectively [11]. The ATR-IR spectrum of the PVA film mineralization is shown in Figure 6B. In addition to the pure PVA membrane, the peak at 1027 cm^–1^ is generated from HA with a phosphate group [33]. As the apatite surface is exposed, the calcium and phosphorus ions in the SBF solution deposited on the membrane surface. These results confirm that the addition of HA and CoHA can significantly enhance the bioactivity effect of PVA membranes.

### 2.8. Biocompatibility

Membrane biocompatibility was evaluated using L929 fibroblasts cultured with PVA and PVA-CoHA nanocomposites in vitro. PVA is a hydrophilic material and has excellent swelling effects, so it will reduce the adhesion ability of cells after swelling, making it difficult to evaluate the cytotoxicity of surface cells.

**Figure 7:**
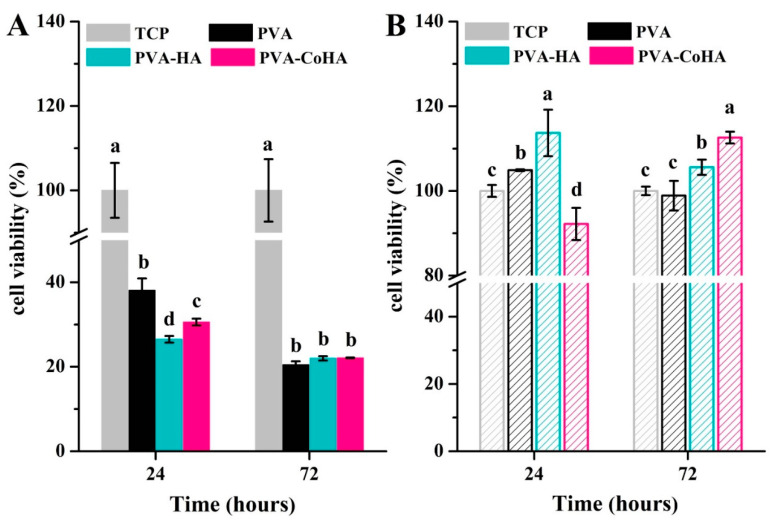
Biocompatibility of the PVA and PVA-CoHA nanocomposite. (**A**) Cells are grown on the surface. (**B**) The cells are cultured using extraction solutions. Means with different letters (a–d) were significantly different (*p* < 0.01, mean ± SD, *n* = 4).

This part of the study is divided into two parts: the membrane surfaces directly in contact with cultured cells, and the extraction of membrane fluid to test the cytotoxicity. As shown in Figure 7A, tissue culture plate (TCP) is significantly higher than the other groups, indicating that cells in the membrane have less desirable surface adhesion. After 24 h, cell attachment for PVA was significantly higher than for the other groups. Seventy-two hours later, the number of PVA-HA and PVA-CoHA cells was not significantly different than for pure PVA. Affixed experiments show that PVA membrane surface cells have poor attachment. For wound dressings however, this phenomenon means that the tissue surrounding the wound is less likely to stick with the dressing and will cause secondary damage.

As to whether the reduction of cells is caused by the release of cobalt ions, we do not know, so the second part uses the membrane material extraction solution for cytotoxicity testing according to the method of ISO 10993-12. The results of the cell culture with the membrane extraction solution are shown in Figure 7B. After the initial 24 h exposure, the results of PVA-HA were significantly higher than for all other groups, and the CoHA groups grow slowly. After 72 h, PVA-CoHA was significantly higher than TCP and pure PVA, but did not differ significantly from the HA groups. The extraction results confirm that the PVA composite membrane has no obvious cytotoxicity. Notably, the release of cobalt ions slows cell growth when cells are initially attached, but it aids cell growth in the latter part of cell proliferation.

### 2.9. Antibacterial Ability

**Figure 8:**
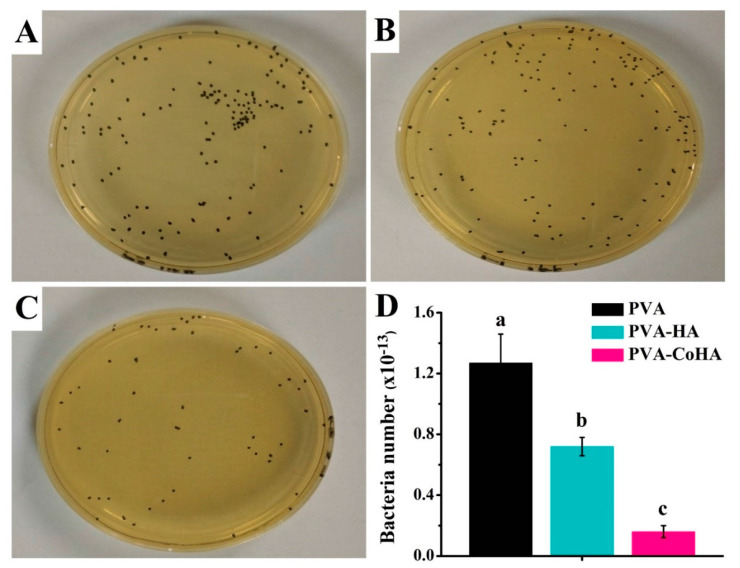
Evaluation of the antibacterial activity of (**A**) PVA, (**B**) PVA-HA and (**C**) PVA-CoHA nanocomposites against *E. coli*. (**D**) Bacteria adhesion on PVA and PVA-CoHA nanocomposites. Means with different letters (a–c) were significantly different (*p* < 0.01, mean ± SD, *n* = 4).

Due to slower recovery in diabetic traumas, wounds are vulnerable to invasion by surrounding bacteria. Therefore, current wound dressings still add antibacterial agents or nanoparticles to achieve antibacterial effects. Through this method, the wound is protected from infection by bacteria on the periphery. In this study, *E. coli* was used to evaluate the antibacterial ability of the membrane. The sample surface of each *E. coli* test was added to a new medium, and 100 λ bacteria was extracted to a petri dish-coated plate for 17 h growth (Figure 8A–C). The number of colonies on a labelled petri dish (*n* = 3) and single factor analysis of variance (ANOVA) were used to assess the statistical significance of the results in Figure 8. That PVA-CoHA group eminently reduced the bacteria viability almost by 87.4% compared to the PVA group (control). The results show that PVA-CoHA has a good antibacterial effect (Figure 8D). Previous studies have also shown that CoHA particles can inhibit the growth of bacteria through the release of cobalt ions [34]. In this study, the CoHA content in PVA-CoHA was very small. Therefore, achieving 100% antibacterial is challenging. Perhaps in the future, the effect of antibacterial ability can be improved by increasing the content of CoHA. Moreover, literature indicates that using Co_3_O_4_ nanoparticles for *E. coli* treatment is effective, with potential for use as antimicrobial agents [35]. This is similar to the results of this experiment and also confirms that the antibacterial effect comes from the addition of cobalt ions. Therefore, PVA added CoHA has the opportunity to inhibit the bacteria around the wound and prevent wound infection.

## 3. Materials and Methods

### 3.1. Synthesis of CoHA

The CoHA powder was prepared by electrochemical deposition [21]. The electrolyte solution was formulated with 42 mM calcium nitrate (Shimakyu’s pure chemical, Osaka, Japan), 25 mM ammonium dihydrogen phosphate (Showa, Tokyo, Japan), and 7.98 mM cobalt chloride (Shimada chemical works, Tokyo, Japan) in de-ionized water. The titanium sheet was a cathode, the stainless-steel sheet was an anode. The electrodeposition procedure was carried out with a direct current power supply (GR-50H10, GICEK, Taipei, Taiwan) and at constant potential by 5.5 V at 328 K for 20 min. After the reaction was completed, rinsed with deionized water and dried, the powder was removed from the surface and collected.

### 3.2. Preparation of PVA and Nanocomposite

The PVA used in this study was obtained from Sigma-Aldrich (St. Louis, MO, USA). The PVA has a molecular weight of 70,000–110,000 g/mole and hydrolysis grade of 98.5%. The homogeneous PVA solution was prepared by adding 1.485 g of PVA in 10 mL of distilled water and stirring at 85 °C for 30 min. Then, 15 mg of CoHA (or HA) powder was then added to the PVA solution and stirred for 24 h at room temperature. The above solution was poured into a polytetrafluroethylene mold (90 mm in diameter) and placed in a fume hood until the solvent evaporated.

### 3.3. Characterization of PVA and Nanocomposite

The surface morphology of the PVA and PVA/CoHA nanocomposite was examined by field-emission scanning electron microscopy (FESEM) (JSM-6700F, JEOL, Tokyo, Japan). Phase indication in samples was confirmed by X-ray diffraction (XRD) (Miniflex II, Rigaku corporation, Tokyo, Japan), operating at 30 kV with Cu-Kα radiation within the scanning range of 10−70° (2θ) and a scanning speed of 4°/min. Infrared spectra of PVA and composite were recorded using a Fourier transform infrared (FTIR) system (FTIR-8000, Shimadzu, Tokyo, Japan) in the spectral range of 650–4000 cm^−1^ with a spectral resolution of 2 cm^−1^, using the attenuated total reflection (ATR) mode. Tensile tests of the PVA and PVA nanocomposites were carried out with universal testing machine (GF-AI-7000M, GO TECH, Taichung, Taiwan) instruments by applying a 10 N load cell at a crosshead speed of 10 mm/min. All samples were cut with a dumbbell-shaped mold 40 mm long and 20 mm wide. The average value of tensile properties was obtained from the results of 3 tests.

### 3.4. Thermal Properties

The thermos-gravimetric analysis (TGA) was carried out on the PVA and PVA nanocomposites using a Q500, TA instrument analyzer (New castle, DE, USA). The samples were heated from 30–600 °C at a heating rate of 10 °C/min under nitrogen, with a nitrogen flow rate of 30 mL/min. The differential scanning calorimetry (DSC) of samples were measured under N_2_ atmosphere with a Q10, TA instrument thermal analyzer (New castle, DE, USA). Samples for DSC measurements were prepared from several circular pieces cut from the polymer film to a mass of about 10 mg. The temperature range studied was 30–400 °C. The heating/cooling rate was 10 °C/min. The degree of inside crystallinity (*X*c) of the PVA membrane was calculated by the following well Equation (1) [11]:

(1) $$X_{C}=\frac{\Delta Hm}{W\times \Delta H_{0}}\times 100,$$

where ∆*H*_0_ is the apparent enthalpy of crystallization, ∆*H*_m_ is the extrapolated value of the enthalpy corresponding to the melting of a 100% crystalline sample (138.6 J/g).

### 3.5. Swelling Behavior

The swelling was measured by immersing the samples in phosphate buffer saline (PBS). All samples were dried in an incubator which was maintained at 37 °C until no change of mass was observed. After, the excessive water on the surface was removed with filter paper. The fully swollen samples were again weighed. The swelling ratio can be calculated as a function of time as swelling ratio (%) = [(W_w_−W_d_)/W_d_] × 100%, where W_d_ is the weight in the dry state of a sample and W_w_ is the weight in the swollen state of the sample.

### 3.6. Free Radical Scavenging Ability

The 2,2-diphenyl-1-picrylhydrazyl (DPPH) was used to evaluate the free radical scavenging ability of PVA and PVA nanocomposites [11]. A control of distilled water (1 mL) or 1 mL of deionized water containing PVA and PVA nanocomposites (30 × 10 mm^2^) was added to 3 mL of DPPH in methanol and left to stand for 90 min. Absorbance of the reaction mixture was then measured at 515 nm with an ultraviolet–visible spectrophotometer (Helios Zeta, Thermo, Waltham, MA, USA). Free radical scavenging ability effect is determined by the following equation: scavenging ratio (%) = [1 − (absorbance of test sample/absorbance of control)] × 100%.

### 3.7. Hydrophilicity Test

The hydrophilicity and hydrophobicity were evaluated through the contact angle test of the material surface. The surface hydrophilicity of the PVA and PVA nanocomposites was evaluated by a contact angle meter (CA-D, Kyowa interface science, Tokyo, Japan). The static contact angle was determined at 25 °C by employing drops of PBS. The quantitative titration of the water drop used was 4 µL and the time was 1 and 60 s. After that, the image was analyzed using the Drop-analysis software attached to Image J for contact angle analysis.

### 3.8. Bioactivity

Simulated body fluid (SBF) was prepared with ionic concentrations nearly equal to those of human blood plasma (K^+^ 5.0, Na^+^ 142.0, Ca^2+^ 2.5, Mg^2+^ 1.5, Cl^−^ 103.0, HCO_3_^−^ 4.2, HPO_4_^2−^ 1.0, and SO_4_^2−^ 0.5 mM; pH 7.42). Samples were pre-cut into square shapes (10 mm × 20 mm) and then immersed in 10 mL of SBF solution in a plastic container, which was tightly closed and kept at 37 °C. The samples were removed after seven days, rinsed with distilled water and lyophilized by a freeze-drying device (FDU-1200, EYELA, Tokyo, Japan) for 24 h [11].

### 3.9. Biocompatibility

To evaluate the biocompatibility, 100 μL polymer solution was coated on a circular glass substrate (15 mm in diameter) and dried. Mouse-derived fibroblasts cell line (L929) was maintained in Dulbecco’s modified eagle medium (DMEM) and supplemented with 10% fetal bovine serum (FBS) at 37 °C in a 5% CO_2_ incubator. Before cell seeding, all samples were sterilized with UV radiation for 1 h. The sterilized samples were placed into 24-well culture plates and seeded with a cell suspension with a cell density of 5 × 10^4^ cells/mL, followed by culturing for 24 and 72 h at 37 °C in 5% CO_2_ incubator. After incubation, samples were rinsed with PBS, followed by incubation in a culture medium containing 1 mL MTT reagent for 4 h. After removal of the medium, 0.5 mL of dimethyl sulfoxide (DMSO) was added to the wells. The 0.1 mL from each sample solution was transferred to 96-well plates and the optical density (O.D.) was measured at 563–650 nm. In addition, the membrane extraction in this study followed the ISO 10993-12 standard. PVA and PVA nanocomposite membranes were cut into square shapes (3 cm^2^) and subjected to sterilization by UV light for 30 min. Then, they were immersed in 75% ethanol solution and followed by PBS replacement. The membrane was immersed in DMEM at 37 °C for 24 h. After, immersion in 1 mL DMEM with 10% FBS at 37 °C in a 5% CO_2_ incubator for 24 h. The extraction solution acquired for each membrane was harvested and used in MTT to determine the cytotoxicity.

### 3.10. Antibacterial Ability

PVA and PVA nanocomposite were investigated against *E. coli* as a model Gram-negative bacteria by the colony plate count method in order to quantify the bacterial effect of our system. The *E. coli* were prepared from fresh brain heart infusion (BHI, Becton Drive, Franklin Lakes, NJ, USA) and incubated at 37 °C for 24 h. The BHI containing *E. coli* was dilute to 10^11^ times the original concentration. Disc shapes (5.5 mm in diameter) of sample was placed into a centrifuge plastic tube and 1 mL of bacteria liquid was extracted and co-cultured for 2 h. Then, the sample was removed and placed in the new BHI solution, then agitated for 10 min. The 100 μL was extracted and applied on the BHI Agar (Becton, Dickinson and Company, TX, USA) petri dish before being cultured for 17 h at 37 °C. Finally, the colonies were counted and the results were expressed as percentage reduction rates bacteria number = [α × 10^11^], where α is the number of bacterial colonies.

### 3.11. Statistical Analysis

All data were expressed as mean ± standard deviation (SD) from three repeat samples. The data were analyzed using JMP 13 software (Statistics Analysis System, NC, USA). A one-way ANOVA followed by a Tukey’s HSD post hoc test was used to determine the level of significance, where *p* < 0.05 was considered to be significant.

## 4. Conclusions

In this study, PVA-CoHA nanocomposite membranes show good swelling behavior, antibacterial, and bioactivity. At the same time, increasing the extensibility of the PVA reduces the damage caused by the dressing when pulled. It is expected that the release of cobalt ions in PVA-CoHA will reduce wound infection and reduce the damage caused by epithelial tissue attachment, discharge the bad tissue fluid through the excellent swelling effect, and keep the wound moist to promote healing. The results support our hypothesis that the addition of CoHA is very effective for the biological activity and anti-inflammatory properties of the membrane. Based on the above, the PVA-CoHA composite membrane described in this study can be a potential choice for diabetic traumas dressings. In the future, we will continue to explore the performance of the PVA-CoHA composite membrane on diabetic wound animal models.

## Figures and Tables

**Table 1:** The relative intensities of 1143 cm^−1^ and 1086 cm^−1^ peak in ATR-FTIR spectra of PVA and PVA/CoHA nanocomposites.

Sample	Absorbance (1143 cm^−1^/1086 cm^−1^)	Surface Crystallinity (%)
PVA	0.524	34.4%
PVA-HA	0.544	35.2%
PVA-CoHA	0.550	35.5%

**Table 2:** Thermal properties of PVA and PVA/CoHA nanocomposites.

Sample	T_onset_ (°C)	T_p_ (°C)	Ash (%)	T_c_ (°C)	T_m_ (°C)	△H_m_ (J/g)	X_c_
PVA	113.0	308.3	0	175.5	206.2	40.7	27.1
PVA-HA	118.0	307.2	1.7	187.4	218.5	53.6	36.1
PVA-CoHA	127.8	306.2	3.1	187.2	218.0	61.6	41.5

T_onset_: onset temperature of pyrolysis, obtained from TGA curves at 95% weight; T_p_: peak pyrolytic temperature, obtained from TGA curves at 50% weight. Crystallization temperature (T_c_), melting temperature (T_m_) and heat of fusion of PVA (∆H_m_), obtained from DSC.

**Table 3:** Calcium to phosphate (Ca/P) ratio of PVA and PVA nanocomposites surface.

Group	Ratio	PVA	PVA-HA	PVA-CoHA
**Before Immersion**	Ca/P	N.D.	1.02	0.86
	Ca + Co/P	N.D.	1.02	0.94
**Immersed in SBF** **for 7 Days**	Ca/P	N.D.	1.29	1.40
	Ca + Co/P	N.D.	1.29	1.40
